# Acceptability of an mHealth App Intervention for Persons With Type 2 Diabetes and its Associations With Initial Self-Management: Randomized Controlled Trial

**DOI:** 10.2196/mhealth.8824

**Published:** 2018-05-21

**Authors:** Astrid Torbjørnsen, Milada Cvancarova Småstuen, Anne Karen Jenum, Eirik Årsand, Lis Ribu

**Affiliations:** ^1^ Department of Nursing and Health Promotion Faculty of Health Sciences Oslo Metropolitan University Oslo Norway; ^2^ Institute of Health and Society General Practice Research Unit, Department of General Practice University of Oslo Oslo Norway; ^3^ Norwegian Centre for E-health Research University Hospital of North Norway Tromsø Norway; ^4^ Department of Clinical Medicine Faculty of Health Sciences UiT The Arctic University of Norway Tromsø Norway

**Keywords:** diabetes mellitus, type 2, patient acceptance of health care, acceptability of health care, self-care, mobile apps, smartphone, telemedicine, regression analysis, factor analysis, statistical

## Abstract

**Background:**

Mobile health interventions are increasingly used in health care. The level of acceptability may indicate whether and how such digital solutions will be used.

**Objective:**

This study aimed to explore associations between the level of acceptability of a mobile diabetes app and initial ability of self-management for patients with type 2 diabetes.

**Methods:**

Participants with type 2 diabetes were recruited from primary health care settings to a 3-armed randomized controlled trial in the Norwegian study in the RENEWING HEALTH project. At the 1-year follow-up, 75 out of 101 participants from the intervention groups completed an acceptability questionnaire (The Service User Technology Acceptability Questionnaire). In the randomized controlled trial, the 2 intervention groups (n=101 in total) received a mobile phone with a diabetes diary app, and one of the groups received additional health counseling given by telephone calls from a diabetes specialist nurse (n=50). At baseline, we collected clinical variables from medical records, whereas demographic data and self-management (The Health Education Impact Questionnaire) measures were self-reported. Log data from the use of the app by self-monitoring were registered continuously. Associations between initial ability to self-manage at baseline and acceptability of the diabetes diary app after 1 year were analyzed using linear regression.

**Results:**

We found statistically significant associations between 5 of the 8 self-management domains and *perceived benefit*, one of the acceptability factors. However, when adjusting for age, gender, and frequency of use, only 1 domain, *skill and technique acquisition,* remained independently associated with *perceived benefit*. Frequency of use of the app was the factor that revealed the strongest association with the acceptability domain *perceived benefit*.

**Conclusions:**

Our findings indicate that persons with diabetes may accept the app, despite its perceived benefit being associated with only one of the 8 domains of their initial level of self-management.

**Trial Registration:**

ClinicalTrials.gov NCT01315756; https://clinicaltrials.gov/show/NCT01315756 (Archived by WebCite at http://www.webcitation.org/6z46qPhWl)

## Introduction

### Background

Self-management is important for persons with chronic illnesses to maintain their own health. Health care providers should engage in self-management support when there is a need for assistance to manage health challenges [1].Both self-management education and support are reported to improve metabolic control as measured by glycated hemoglobin (HbA_1c_) levels for persons with type 2 diabetes [2]. Furthermore, mobile health (mHealth) interventions developed for diabetes self-management have shown some effects, although little is known about the full potential benefit of using mobile diabetes apps [3-7]. Successful use of mHealth tools and services requires an active user and cooperation with health professionals [4]. Use of mHealth often includes the possibility of sharing data between health professionals and their patients with diabetes, which could enhance the support to improve their self-management [6,7].

The acceptability of the provided mobile-based technology is important for their use and for its implementation into practice [8]. However, only sparse knowledge exists about factors that make mobile technology acceptable for persons with type 2 diabetes [9-11]. Findings from the Whole System Demonstrator (WSD) study indicate a positive association between self-management and higher levels of acceptability [12]. Other studies have found associations between satisfaction with the device and improved diabetes management [9], but to our knowledge, little is known about the associations between the acceptability of an app and the initial ability to self-manage one’s own health, before introducing the app. We hypothesized that a person with a high degree of self-management at baseline would have the skills and confidence to accept and implement the use of available technical tools in self-care.

### Objectives

The aim of this study was to explore associations between the initial ability of self-management and the level of acceptability of a mobile diabetes app.

## Methods

### Participants and Setting

The study sample in this study consisted of participants from the Norwegian randomized controlled trial (RCT) of the European Union project RENEWING HEALTH, which has been described in detail elsewhere [13-15]. Participants were persons with type 2 diabetes mainly recruited through general practitioners between March 2011 and September 2012. Eligibility criteria were (1) adults aged ≥18 years with type 2 diabetes, (2) HbA_1c_ levels ≥7.1%, and (3) capacity to use the equipment and to fill in questionnaires in Norwegian. The study was a 3-armed RCT with 2 intervention groups and 1 control group. Both intervention groups (n=50+51) received a mobile phone with a diabetes diary app developed at the Norwegian Centre for E-health Research [16] and a blood glucose meter (OneTouch Ultra Easy from LifeScan Inc. West Chester, PA, USA), equipped with an adapter for enabling Bluetooth communication (Polytel GMA from Polymap Wireless). One of the intervention groups received additional health counseling through telephone calls by a diabetes specialist nurse for the first 4 months of the study. At the 1-year follow-up, 75 out of 101 participants from the intervention groups completed an acceptability questionnaire (The Service User Technology Acceptability Questionnaire) after having finished the study.

Both intervention groups received the training needed to manage the mobile phone and the app provided by a team of researchers and assistants in meetings with the participants, in addition to a technical support telephone service available at daytime.

Despite the eligibility criteria “capacity to use the app, some participants expressed a lack of motivation or capacity to learn to use the app. Some therefore received additional training in face-to-face meetings with the technical supporters or others in the research team. In the health counseling intervention group, the diabetes specialist nurse focused on diabetes self-management and motivation, and at the same time, when needed, encouraged the participants to use the app [13,14]. Currently, we report findings from the 2 intervention groups that were assessed at the 1-year follow-up; in total, 75 of the originally enrolled 101 participants completed the self-report questionnaires (response rate; 74.3%).

### Measures

#### The Service User Technology Acceptability Questionnaire

The Service User Technology Acceptability Questionnaire (SUTAQ) was developed, designed, and psychometrically evaluated for the WSD study, a large telehealth study performed in England. The 22 items aim to measure the users’ beliefs and perceptions of the equipment. An expert panel of researchers and clinicians developed the questionnaire. Factor analysis from the original WSD study reported 5 domains: *perceived benefit, privacy and discomfort*, *care personnel concerns*, *kit as a substitution*, and *satisfaction* [12]. The answers to the statements for each item were rated on a Likert scale from 1 to 6, ranging from strongly agree to strongly disagree [12]. The psychometric evaluation of the Norwegian language version of SUTAQ is reported elsewhere, and the factor analyses only confirmed the domains *perceived benefit* and *care personnel concerns* [17].

**Table 1:** Sample characteristics at baseline for those who responded at 1-year follow-up (Service User Technology Acceptability Questionnaire, n=75).

Variables			Median	Range
Age (years)			59	35-80
Diabetes duration^a^ (years)			9	1-36
**HbA_1c_^b^, %**				
	Baseline		7.8	7.1-12.4
	1-year follow-up^c^		7.6	5.6-13.0
**Health Education Impact Questionnaire domains**				
	Health-directed activity		2.75	1.00-4.00
	Positive and active engagement in life		3.20	1.60-4.00
	Emotional distress		3.00	1.17-4.00
	Self-monitoring and insight		3.00	2.33-3.83
	Constructive attitudes and approaches		3.00	1.80-4.00
	Skill and technique acquisition		3.00	2.00-4.00
	Social integration and support		3.00	2.00-4.00
	Health service navigation		3.00	2.00-4.00

^a^Missing from baseline: n=6.

^b^HbA_1c_: glycated hemoglobin.

^c^Missing from baseline: n=2.

#### The Health Education Impact Questionnaire

The Health Education Impact Questionnaire (heiQ) was developed in Australia [18] to measure self-management after participation in education and or self-management support interventions for persons with chronic diseases. This questionnaire has later been adapted for multiple settings. In addition, Osborne has suggested new ways of use, such as incorporation of the instrument, or some of the scales, into standard assessment and as a care planning tool [1]. Each of the 40 items is rated on a Likert scale from 1 to 4, from “strongly disagree” to “strongly agree.” They are organized into 8 domains as listed in Table 1, with 4 to 6 items in each domain. For all domains, high scores indicate a high level of self-management abilities, except for *emotional distress*, where high scores reflect high distress. The heiQ questionnaire has been validated in a Norwegian primary health care context in patients with different chronic conditions, including diabetes [19]. In this study, we used heiQ data from baseline measures before any intervention to investigate the users’ initial ability to self-manage.

### Log Data, High and Low Frequency of Use

The researchers in the team defined high frequency of use as ≥5 blood glucose measurements and ≥50 keystrokes in the app each month for at least 6 months of the 1-year intervention to differentiate between participants who used the app regularly, sporadically, or did not use the app. The app enabled registration of blood glucose level, diet and physical activity, setting of goals, and gave access to a diabetes-specific dictionary. The Bluetooth technology enabled automatic sending of blood glucose values from the blood glucose meter to the app. Diet and physical activity data were self-reported and entered manually into the app through graphical user interface. Keystrokes were registered for use of all the graphical user interface functionalities. Measurements of blood glucose, diet, and physical activity that were recorded with the app were sent to a secure server continuously during the study.

### Demographic and Clinical Data

Demographic data such as age, gender, education, diabetes duration, and any comorbidities were self-reported through questionnaires at baseline. HbA_1c_ baseline values were obtained from the general practitioners’ medical records.

### Statistical Analysis

Sample characteristics are presented as counts and percentages for categorical variables and as median and range for continuous variables. Differences between the intervention groups and between the participants lost to follow-up and the responders were assessed using Pearson chi-square test for pairs of categorical data and the nonparametric Mann-Whitney Wilcoxon *U* test for continuous data. We modeled associations between initial ability to self-manage (heiQ) and equipment acceptability (SUTAQ) with univariate linear regression models, and thereafter adjusted for possible confounders such as age, gender, and frequency of use in multiple models. *P* values <.05 were considered statistically significant. All tests were two-sided. All analyses were performed using IBM SPSS Statistics (v 23; IBM Corp, Armonk, NY, USA).

### Ethics

The Norwegian Regional Committees for Medical and Health Research Ethics approved the study, and the participants signed an informed consent when they entered. In addition, we performed risk analysis before start of the study.

## Results

### Sample Characteristics

Of the 75 participants analyzed, 42 (56%) were female, the median age was 59 years (age range 35-80 years), 37 (49%) had 12 years or more of education, only 10 (13%) had no comorbidities, and the median diabetes duration was 9 years (range 1-36 years). Almost half of the participants, that is, 48% (36/75), were high-frequency users of the diabetes diary app (Tables 1 and 2). No statistically significant differences in self-reported acceptability (SUTAQ) of the equipment or in baseline measures between the 2 intervention groups were revealed. Furthermore, between the participants lost to follow-up at 1 year (nonresponders) and the remaining responders in the interventions groups, there were no differences in baseline values regarding age, gender, education, diabetes duration, or HbA_1c_; however, we did find a difference in the frequency of use of the app. According to the log data, only one of the participants lost to follow-up used the app frequently. Overall, heiQ baseline values were in the slightly higher ranges of possible values for all the measured items (Table 1).

### Associations Between Self-Management Assessed With Health Education Impact Questionnaire and Acceptability Measured With Service User Technology Acceptability Questionnaire

We explored the 2 acceptability factors *perceived benefit* and *care personnel concerns*, which were the only domains of the original scale that were confirmed by the factor analysis [17]. The domain *perceived benefit* (SUTAQ) was significantly associated with 5 of the 8 heiQ (self-management) domains at baseline (Table 3).

In addition, our data revealed a significant crude association between gender and *perceived benefit*, where men experienced more benefit from the app than women (estimated beta=−.57, 95% CI −1.05 to −0.09, *P=*.02). Moreover, an association was revealed between gender and frequency of use, where 69% (25/36) of the high-frequency users were men (*P*=.02).

Furthermore, linear regression models confirmed that frequency of equipment use was the factor that was strongest associated with *perceived benefit* (SUTAQ), even when controlled for all of the heiQ domains separately, as well as for age and gender. Only the heiQ domain *skill and technique acquisition* remained associated with *perceived benefit* when adjusted for age, gender, and frequency of use (Table 3). No association was found between initial ability to self-manage (heiQ) and the SUTAQ domain *care personnel concerns* (results not shown).

**Table 2:** Sample characteristics at baseline, 1-year follow-up responders (Service User Technology Acceptability Questionnaire); n=75.

Variables		n (%)
**Gender**		
	Female	42 (56)
	Male	33 (44)
**Education**		
	<12 years	38 (51)
	12 years	7 (9)
	>12 years	30 (40)
**Comorbidities**		
	0	10 (13)
	1-2	51 (68)
	≥3	14 (19)
**Use of app, log data^a^**		
	Low frequency of use	37 (49)
	High frequency of use	36 (48)
**Familiar with technology**		
	Familiar with use of computer	69 (92)
	Familiar with use of mobile phone	75 (100)

^a^Missing from baseline: n=2 (3%).

**Table 3:** Linear regression and crude and adjusted values (adjusted for age, gender, and frequency of use), dependent variable *perceived benefit* (Service User Technology Acceptability Questionnaire).

heiQ^a^ domains	Crude		Adjusted (age, gender, and frequency of use)	
	Estimated beta (95% CI)	*P* value	Estimated beta (95% CI)	*P* value
Health-directed activity	.44 (0.11 to 0.78)	.01	.31 (−0.03 to 0.64)	.07
Positive and active engagement in life	.40 (−0.19 to 0.98)	.18	.17 (−0.40 to 0.73)	.56
Emotional distress	.37 (−0.03 to 0.78)	.07	.20 (−0.20 to 0.59)	.32
Self-monitoring and insight	.89 (0.05 to 1.74)	.04	.53 (−0.30 to 1.36)	.21
Constructive attitudes and approaches	.45 (−0.01 to 0.91)	.06	.29 (−0.16 to 0.74)	.21
Skill and technique acquisition	.74 (0.17 to 1.32)	.01	.60 (0.03 to 1.17)	.04
Social integration and support	.69 (0.21 to 1.18)	.005	.37 (−0.15 to 0.90)	.16
Health service navigation	.64 (0.14 to 1.14)	.01	.40 (−0.11 to 0.92)	.12

^a^heiQ: Health Education Impact Questionnaire.

## Discussion

### Principal Findings

We explored associations between initial ability to self-manage and equipment acceptability using the 2 acceptability factors *perceived benefit* and *care personnel concerns*, which were the only 2 domains confirmed in the factor analysis. As hypothesized, we found a linear relationship between higher self-management and a positive experience of the mobile diabetes app as being beneficial for health care. However, after adjusting for age, gender, and frequency of use, this association was no longer statistically significant, except for the domain *skill and technique acquisition*. Furthermore, according to our findings, the use of the app turned out to have the strongest association with app acceptability.

The SUTAQ domain *perceived benefit* contains statements regarding improved health, increased involvement in health treatment, and the use of the app and the equipment as a supplement to usual care. In contrast to what we assumed, the initial ability to self-manage did not seem to be associated with acceptability; however, the participants who used the mobile phone app reported benefits of the app independent of the level of perceived self-management before its use. There are several barriers for persons with type 2 diabetes concerning the use of digital tools in their treatment. These barriers could be related to the potential user, to the technology, or to the health care offered [20-22]. Technical difficulties [9] and technological illiteracy [20,21], in addition to low health literacy [23,24], are associated with less engagement with technology for persons with type 2 diabetes. We did not find initial low self-management to be a barrier in our study, as our nonresponders had similar levels of all items of heiQ. However, it may not have been a coincidence that the domain that remained statistically significant in the analyses was skills to manage symptoms, which include skills to make use of equipment [1,18]. The participants were motivated to use the technology when they volunteered to enter the study, and inability to use the technology was an exclusion criterion. We can speculate that a lower self-management could have been a barrier at an earlier stage with regard to showing interest in the study. This is a limitation to the generalizability of our findings as all our participants scored relatively high on heiQ. In contrast to our findings, Hirani et al found an association between several of the heiQ domains and the SUTAQ domains, which indicates that those who accepted the intervention reported higher levels of self-management [12]. However, Hirani et al did not report the baseline values of heiQ. Therefore, it remains unclear whether their findings reflect a change in self-management during the use of the digital tools, where use of the tools enhances self-management skills and attitude, or whether the level of self-management was unchanged from baseline.

### Strengths and Limitations of the Study

The participants’ technical literacy, which was an inclusion criterion in our study, could bias our findings, as all participants were able to manage the equipment at some level, increasing the probability of acceptability. Nevertheless, we experienced a noticeable diversity in technical skills, and we gave technical support when needed through the study, implying that participants with different levels of technical experience and preferences were included, although persons with lack of technical skills were not eligible. It would have strengthened our study if we had measured the initial level of technological skills in more detail than only their experiences with mobile phones and personal computers. All the participants had previous experiences with use of mobile phone, yet not necessarily smartphones. Another possible limitation is the definition of frequency of use. It is difficult precisely to define an anticipated use because of between-persons differences in needs and stage of progression of their disease. Our definition of use aimed to differentiate between the participants’ use of the app regularly and the ones who had sporadic or no use of the app.

In addition, it would have been interesting to know their level of motivation to enter the study, as we do not know much about who were initially eager to try new technology or whether they attended for other reasons. However, we have performed a qualitative study with interviews at the end of the RCT when the participants left the study and gained more in-depth knowledge about the participants’ acceptability. These findings have recently been corroborated (A Torbjørnsen et al, unpublished data, May 2018).

In our analysis, we pooled together 2 heterogeneous groups of participants testing the app, with 1 group receiving initial health counseling. Potentially, the group receiving health counseling could have responded differently to the acceptability questionnaire than the intervention group that only had the equipment with the app. As an example, the health counseling group could have reported higher acceptability caused by enhanced access to health care and technical support; to the contrary, the health counseling could add a burden, and exhausted the participants, and thus, resulting in lower acceptability by some. As we did not find any differences between the groups, neither for acceptability nor for other measures, we pooled the 2 groups. We have previously discussed the effect and intensity of the health counseling [14,15].

### Implications for Future Research and Clinical Practice

Our findings suggest that use of a diabetes diary app could be acceptable regardless of initial ability to self-manage, as the crude correlation between the 2 scales disappeared when adjusting for age, gender, and frequency of use, except for the domain *skill and technique acquisition*. Further research on which factors may influence the use and benefit of an mHealth solution would be of interest.

## Appendix

Multimedia Appendix 1 CONSORT‐EHEALTH checklist (V.1.6.1).

## Abbreviations

HbA _1c_: glycated hemoglobin
heiQ: Health Education Impact Questionnaire
mHealth: mobile health
RCT: randomized controlled trial
SUTAQ: Service User Technology Acceptability Questionnaire
WSD: Whole System Demonstrator
